# Chemoselectivity-independent Cu-mediated coupling to construct the hydroquinoline skeleton of symbioimine

**DOI:** 10.1038/s41598-021-03448-9

**Published:** 2021-12-15

**Authors:** Rie Fujita, Kengo Hanaya, Takeshi Sugai, Shuhei Higashibayashi

**Affiliations:** Faculty of Pharmacy, Keio University, 1-5-30 Shibakoen, Minato-ku, Tokyo, 105-8512 Japan

**Keywords:** Synthetic chemistry methodology, Natural product synthesis

## Abstract

Construction of the hydroquinoline skeleton of symbioimine by Cu-mediated *N*-alkenylation or *O*-alkenylation of an allyl aminoalcohol, in which either chemoselectivity could lead to the target compound, was investigated. *O*-alkenylation followed by Claisen rearrangement was favored with high selectivity under a ligand-free condition. Subsequent intramolecular condensation furnished the hydroquinoline skeleton of symbioimine.

## Introduction

**Scheme 1:**
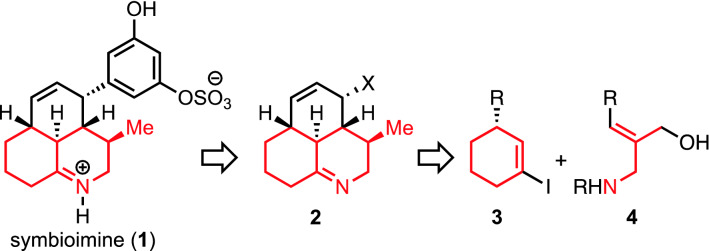
Symbiomine (**1**) and its synthetic plan through a tricyclic core **2** from two components **3** and **4**. The hydroquinoline skeleton is highlighted in red.

**Scheme 2:**
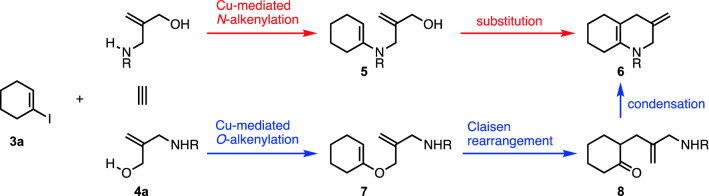
Chemoselectivity-independent approach to construct the hydroquinoline skeleton of symbioimine (**1**).

In 2004, Uemura and coworkers reported the isolation of symbioimine (**1**) (Scheme 1) from dinoflagellate *Symbiodinium* sp. having a symbiotic relationship with *Amphiscolops* sp.^1^. Symbioimine (**1**) inhibits osteoclastogenesis in RAW264 cells (EC_50_ = 44 μM) without showing toxicity (100 μg/mL)^1–3^. It also shows cyclooxygenase-2 (COX-2) inhibitory activity (10 uM)^2,3^. These biological activities suggest its application for antiresorptive and anti-inflammatory agents. Symbioimine (**1**) possesses a tricyclic skeleton with an aromatic substituent, in which an iminium cation and a sulfonium anion form a zwitterionic pair. While several research groups have reported the synthesis of **1**, all syntheses employed an intramolecular Diels–Alder reaction of dienes possessing an aromatic substituent for the construction of the skeleton^4–10^, based on the proposed biosynthesis by Uemura^3^. However, this approach is not amenable to the syntheses of derivatives of **1** such as those with different aromatic groups for the improvement of the pharmacological activity, since the aromatic substituent was introduced at the early stage of the syntheses before the construction of the tricyclic core. Despite the attractive biological activities, the structure–activity relationship of **1** has not been elucidated. We planned a new synthetic approach for the synthesis of **1** as well as derivatives with different aromatic substituents, in which the aromatic rings are introduced at the late stage of the synthesis after the construction of the tricyclic core **2** (Scheme 1). Toward the synthesis, we devised a route to construct the hydroquinoline moiety of **2** by the coupling between iodocyclohexene **3** and allyl aminoalcohol **4** (Scheme 1). The Cu-mediated *N*-alkenylation^11–13^ to enamine **5** followed by a substitution reaction was expected to afford the hydroquinoline skeleton **6** (Scheme 2). However, Cu-mediated *O*-alkenylation could compete with *N*-alkenylation^12,14–17^. Generally in a chemoselective coupling reaction with a substrate having two reactive sites, one coupling with one site affords the desired product, while another coupling with another site does not lead to the desired product. Therefore, the success of the synthesis is dependent on the achievement of the desired chemoselectivity. In our planned synthesis, however, the *O*-alkenylation to **7** followed by Claisen rearrangement^18^ to **8** and condensation was also expected to afford the desired hydroquinoline skeleton **6** (Scheme 2). Thus, either chemoselectivity is acceptable to furnish the target compound, namely, we have devised a chemoselectivity-independent approach. To verify this concept, we investigated the synthesis of the hydroquinoline skeleton **6** of symbioimine (**1**) through the Cu-mediated coupling of alkenyl iodide **3a** and allyl aminoalcohol **4a**.

## Results and discussion

**Scheme 3:**
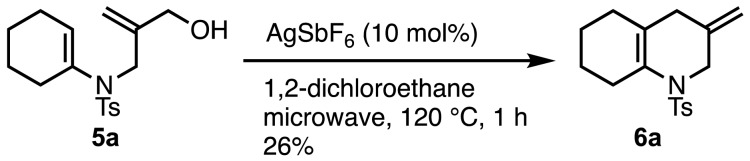
Conversion of enamine **5a** to hydroquinoline **6a**.

Sulfonamide **4aa** (R = Ts) of the allyl aminoalcohol was chosen as the coupling partner owing to the stability of the target enamine, which was prepared according to the literature from ethyl 2-(hydroxymethyl)-2-propenoate in three steps^19,20^. While Cu-catalyzed selective *N*-arylation or *O*-arylation conditions of aminoalcohols have been reported to date^12,14–17^, a selective arylation or alkenylation between sulfonamides and alcohols has not been investigated. After initial screening of the coupling conditions between alkenyl iodide **3a** and sulfonamide **4aa**, CuI (1.0 eq.) and *N*,*N*′-dimethylethylenediamine (2.0 eq.) were employed, and bases (2.5 eq.) and solvents were screened under reflux conditions for 19–24 h with 2.0 eq. of **3a** (Table 1)^21–23^. In entry 1 using K_3_PO_4_ in CH_3_CN, a mixture of enamine **5a** through *N*-alkenylation, ketone **8a** through *O*-alkenylation followed by Claisen rearrangement, imine **9a** through *N*-alkenylation followed by aza-Claisen rearrangement, and several products through both *N*- and *O*-alkenylations and Claisen rearrangements were obtained. The yields of **5a**, **8a**, and **9a** were 14%, 23%, and 7% with recovery of 49% of **4aa**, respectively. In entries 2–5, KO^*t*^Bu, CsOAc, K_2_CO_3_, and Cs_2_CO_3_ were used as bases in CH_3_CN. In entry 5 using Cs_2_CO_3_, the yield of **5a** and the chemoselectivity were slightly improved, giving **5a** in 26% yield. In entries 6–8, DMF, DMSO, and toluene were used as solvents. However, the yield of **5a** and the chemoselectivity were not improved. Despite the low yield, enamine **5a** was converted to hydroquinoline **6a** in 26% yield by an acid-catalyzed substitution reaction^24^ (Scheme 3).

**Table 1:** Screening for Cu-mediated *N*-alkenylation.


Entry	Base	Solvent	Yield (%)^a^			
			4aa	5a	8a	9a
1	K_3_PO_4_	CH_3_CN	49	14	23	7
2	KO^*t*^Bu	CH_3_CN	30	7	17	0
3	CsOAc	CH_3_CN	56	12	Trace	0
4	K_2_CO_3_	CH_3_CN	79	10	0	0
5	Cs_2_CO_3_	CH_3_CN	19	26	13	0
6	Cs_2_CO_3_	DMF	19	21	10	0
7	Cs_2_CO_3_	DMSO	38	13	13	0
8	Cs_2_CO_3_	toluene	18	17	12	0

^a^Yields were determined by ^1^H NMR spectroscopy using CH_3_NO_2_ as an internal standard.

**Scheme 4:**
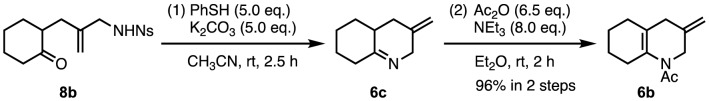
Conversion of ketone **8b** to hydroquinoline **6**.

Although the target hydroquinoline skeleton was constructed, the yield of **6a** was too low for application to the synthesis of symbioimine (**1**). The primary problem was the poor selectivity and yield of the coupling between **3a** and **4aa**. Buckwald et al*.* reported that an electron-rich anionic ligand and an electron-poor neutral ligand favored *N*-arylation and *O*-arylation, respectively, but the ligand-assisted conditions were not effective for coupling chelating aminoalcohols with fewer methylene groups^14^. Instead, it was reported that ligand-free conditions were effective for selective *N*-arylation of chelating aminoalcohols, in which the selectivity depended on the solvent^14,15^. Buckwald et al*.* reported the selective *N*-arylation of ethanolamine in DMF and 3-piperidinol in CH_3_CN in 40:1–25:1 ratios and the *O*-arylation of 3-amino-1-propanol in toluene and 3-piperidinol in THF in a 1:2 ratio^14^. Chan et al*.* also reported that *N*- and *O*-arylations of 3-amino-1-propanol occurred in DMF with a 13:3 ratio and in toluene with a 4:15 ratio^15^. Thus, we investigated the coupling of **3a** and **4aa** under ligand-free conditions in several solvents (Table 2). In CH_3_CN (entry 1), surprisingly, ketone **8a** through *O*-alkenylation followed by Claisen rearrangement was selectively obtained in 67% yield. In DMF (entry 2) or toluene (entry 3), the yield of **8a** significantly decreased to 26% and 9%. In DMSO (entry 4) or THF (entry 5), **8a** was not formed and **5** was produced in 12% and 19% yields. The coupling between **3a** and **4ab** (R = Ns) in CH_3_CN afforded **8b** in 70% yield (entry 6). Under all conditions, enol ether **7** through *O*-alkenylation was not observed. Density functional theory (DFT) calculations at the PBE0/6–31 + G(d) level of theory showed that the activation barrier for the Claisen rearrangement is 23.4 kcal/mol and ketone **8** (R = SO_2_Ph) is 21.1 kcal/mol more stable than **7**, which is consistent with the experimental results. It is noteworthy that our observed solvent effect on the selectivity of coupling for the sulfonamides of the aminoalcohol was opposite to the reported ones for aminoalcohols and the highly selective *O*-alkenylation was achieved. Since selective *O*-arylation of chelating aminoalcohols had been an unsolved problem^14,15^, the generality of our result is of great interest. With the selective *O*-alkenylation condition in hand instead of *N*-alkenylation, ketone **8b** was converted to hydroquinoline **6b** (Scheme 4). Deprotection of the *o*-nitrobenzenesulfonyl group of **8b** by PhSH and K_2_CO_3_^25^ followed by spontaneous intramolecular condensation furnished hydroquinoline **6c**. Since **6c** was not isolated by silica gel column chromatography owing to the instability to the acidic condition, **6c** was converted to amide, giving hydroquinoline **6b** in 96% yield (2 steps).

**Table 2:** Screening for Cu-mediated *O*-alkenylation and Claisen rearrangement.

Entry	R	Solvent	Yield (%)^a^			
			4a	5	8	9
1	Ts	CH_3_CN	15	0	67 (64)	0
2	Ts	DMF	16	0	26	19
3	Ts	Toluene	25	0	9	0
4	Ts	DMSO	26	12	0	0
5	Ts	THF	33	19	0	0
6	Ns	CH_3_CN	10	0	70 (68)	0

^a^Yields were determined by ^1^H NMR spectroscopy using CH_3_NO_2_ as an internal standard. The numbers in parentheses are isolated yields.

## Conclusions

In summary, construction of the hydroquinoline skeleton of symbioimine was investigated by a chemoselectivity-independent approach employing Cu-mediated *N*-alkenylation or *O*-alkenylation of sulfonamides of an allyl aminoalcohol. While *N*-alkenylation took place in low yield with poor selectivity under ligand-assisted conditions, high yield and selectivity for *O*-alkenylation followed by Claisen rearrangement were achieved with a ligand-free condition. Both products through *N*- and *O*-alkenylation were converted to the target hydroquinoline skeleton. This approach was proven to be promising to synthesize the target skeleton and its application to the synthesis of symbioimine is in progress. In addition, the generality of the selective *O*-alkenylation condition to various aminoalcohols bearing sulfonamide, amide, and carbamate is currently under investigation in our laboratory.

## Methods

### General procedure of Cu-mediated N- and O-alkenylation (Tables 1 and 2)

Copper iodide (CuI) was prepared according to the literature procedure^26^. Purified CuI (38 mg, 0.20 mmol) was suspended in anhydrous solvent (0.10 mL) under an argon atmosphere. Under the conditions in Table 1, *N*,*N*′-dimethylethylenediamine (45 μL, 0.40 mmol) was added to the suspension. To the solution, sulfonamide **4aa** or **4ab** (0.20 mmol) in anhydrous solvent (0.10 mL), base (0.50 mmol), and vinyl iodide **3a** (83 mg, 0.40 mmol) were added at 0 °C. The reaction mixture was stirred under heating for 19–24 h. The reaction mixture was cooled to room temperature and quenched by addition of saturated NH_4_Cl aq. (0.5 mL). The organic layer was separated, and the aqueous layer was extracted with EtOAc (1.0 mL) three times. The combined organic layer was washed with brine, dried over Na_2_SO_4_, and filtered through a cotton plug. The filtrate was concentrated *in vacuo*, and the residue was purified by silica gel column chromatography with hexane/EtOAc (10/1 to 5/1) to isolate **5**, **8** and **9**.

## Supplementary Information

Supplementary Information.
