# Supplementary material for: Chemoselectivity-independent Cu-mediated coupling to construct the hydroquinoline skeleton of symbioimine
Source: Sci Rep. 2021 Dec 15;11:24078. doi: 10.1038/s41598-021-03448-9 (PMC8674349; doi:10.1038/s41598-021-03448-9)
Supplement: Supplementary file 1 — Supplementary Information. [file 41598_2021_3448_MOESM1_ESM.pdf]

**Chemoselectivity-independent Cu-mediated coupling to construct the hydroquinoline skeleton of symbioimine**

Rie Fujita, Kengo Hanaya, Takeshi Sugai and Shuhei Higashibayashi*

*Faculty of Pharmacy, Keio University, 1-5-30 Shibakoen, Minato-ku, Tokyo 105-8512, Japan*

## Table of Contents

1. General Information S1

2. Synthesis and Characterization Data of Compounds

2.1 Preparation of 1-iodocyclohex-1-ene (**3a**) S1

2.2 Preparation of sulfonamides **4a** S2

2.3 Cu-mediated *N*- and *O*-alkenylation S4

2.4 Conversion to hydroquinoline **6** S5

3. $^{1}H$ and $^{13}C$ NMR Spectra S7

4. Theoretical Calculation S15

5. References S16

## 1. General Information

Reagents and solvents for syntheses were commercially purchased and used as received. Air and/or moisture sensitive reactions were carried out by using anhydrous solvents under an argon atmosphere. TLC analysis was performed using Merck TLC Silica gel 60 $F_{254}$. Flash silica gel column chromatography was performed on Wako Wakosil® C-300. IR spectra were recorded on a Jasco FT/IR-4700 spectrometer with ATR PRO ONE in ATR mode using diamond prism. $^{1}$H and $^{13}$C NMR spectra were measured on a Bruker spectrometer at 500 and 126 MHz. $CDCl_3$ was used as a solvent and the residual solvent peaks were used as an internal standard ($CDCl_3$: $^{1}$H NMR: 7.26 ppm; $^{13}$C NMR: 77.0 ppm). High resolution (HR) mass spectra (MS) were measured on JEOL JMS-T100LP using electrospray ionization (ESI). The reactions at high temperature were performed with Organic Synthesizer, EYELA ChemiStation, using aluminum heating block with cooling circulator. Microwave irradiation experiment was carried out with Biotage® Initiator+.

## 2. Synthesis and Characterization Data of Compounds

### 2.1 Preparation of 1-iodocyclohex-1-ene (3a)

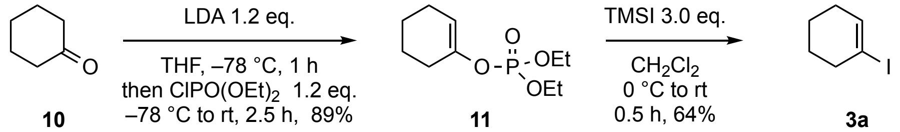


**Scheme S1.** Preparation of 1-iodocyclohex-1-ene (**3a**)

**Cyclohex-1-en-1-yl diethyl phosphate (11)**

Diethyl vinyl phosphate **11** was prepared according to the literature procedure.[1] A solution of lithium diisopropylamide (LDA) prepared from *i*-$Pr_2NH$ (8.5 mL, 60 mmol) and *n*-BuLi (1.4 M in hexane, 43 mL, 60 mmol) in anhydrous THF (40 mL) was cooled to -78 ºC under an argon atmosphere. Cyclohexanone **10** (4.90 g, 50 mmol) in anhydrous THF (10 mL) was added dropwise to the solution, and the mixture was stirred for 1 hour at the same temperature. Diethyl phosphorochloridate (10.4 g, 60 mmol) in anhydrous THF (10 mL) was added to the mixture. After stirred for 2.5 hour at room temperature, the reaction was quenched by addition of saturated $NH_4Cl$ aq. (50 mL) at 0 ºC, and the organic layer was separated. The aqueous layer was extracted with EtOAc (50 mL) twice. The combined organic layer was washed with brine, dried over $Na_2SO_4$, and filtered through a cotton plug. The filtrate was concentrated *in vacuo*, and the residue was purified by silica gel column chromatography with hexane/EtOAc (5/1) to afford **11** (10.4 g, 89% yield) as slightly yellow oil. The $^{1}$H NMR spectrum was identical to that in the literature.[1] $^{1}$H NMR ($CDCl_3$): δ 5.47 (m, 1H), 4.18-4.11 (m, 4H), 2.19 (m, 2H), 2.08-2.07 (m, 2H), 1.73-1.68 (m, 2H), 1.57-1.52 (m, 2H), 1.36-1.33 (m, 6H) ppm.

**1-Iodocyclohex-1-ene (3a)**

Vinyl iodide **3a** was prepared according to the literature procedure.[2] Diethyl vinyl phosphate **11** (10.4 g, 45 mmol) was dissolved in anhydrous $CH_2Cl_2$ (89 mL) under an argon atmosphere, and the solution was cooled

to 0 ºC. TMSI (26.7 mL, 134 mmol) was added to the solution, and the mixture was stirred at room temperature for 30 min. The reaction was quenched by addition of saturated $Na_2S_2O_3$ aq. (100 mL) and saturated $NaHCO_3$ (100 mL) at 0 ºC. The organic layer was separated, and the aqueous layer was extracted with $CH_2Cl_2$ (70 mL). The combined organic layer was washed with brine, dried over $Na_2SO_4$, and filtered through a cotton plug. The filtrate was concentrated *in vacuo*, and the residue was purified by silica gel column chromatography with hexane to afford **3a** (5.9 g, 64% yield) as colorless oil. The $^1H$ NMR spectrum was identical to that in the literature.[3] $^1H$ NMR ($CDCl_3$): δ 6.35-6.33 (m, 1H), 2.52-2.48 (m, 2H), 2.11-2.07 (m, 2H), 1.72-1.63 (m, 4H) ppm; $^{13}C$ NMR ($CDCl_3$): 137.5, 96.9, 39.4, 29.0, 25.3, 20.9 ppm; $^{13}C$ NMR ($CDCl_3$): δ 137.5, 96.9, 39.4, 29.0, 25.3, 20.9 ppm.

## 2.2 Preparation of sulfonamides 4a

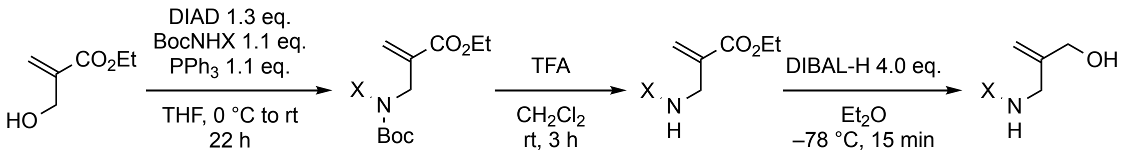


**12** **13a** (X = Ts) **13b** (X =Ns) **14a** (X = Ts) **14b** (X =Ns) **4aa** (X = Ts) **4ab** (X =Ns)

**Scheme S2.** Preparation of sulfonamides **4a**

**Ethyl 2-[{(*N*-(tert-butoxycarbonyl)-4-methylphenyl)sulfonamido}methyl]acrylate (13a)**

Acrylate **13a** was prepared according to the literature procedure.[4] Ethyl 2-(hydroxymethyl)acrylate (**12**) (1.95 g, 15 mmol) was dissolved in anhydrous THF (150 mL) under an argon atmosphere, and the solution was cooled to 0 ºC. $PPh_3$ (4.33 g, 17 mmol) and BocNHTs (4.48g, 17 mmol) were added to the solution. Then, diisopropyl azodicarboxylate (DIAD) (1.9 M in toluene, 10.5 mL, 20 mmol) was added dropwise over 10 min at 0 ºC. After stirring at room temperature for 22 hours, the reaction solution was concentrated *in vacuo*, and the residue was purified by silica gel column chromatography with hexane/EtOAc (9/1) to afford **13a** (5.23 g, 91% yield) as colorless oil. The $^1H$ NMR spectrum was identical to that in the literature.[4] $^1H$ NMR ($CDCl_3$): δ 7.81 (d, $J$ = 8.3 Hz, 2H) 7.31 (d, $J$ = 8.3 Hz, 2H), 6.36 (s, 1H) ppm, 5.75 (s, 1H), 4.71 (s, 2H), 4.23 (q, $J$ = 7.2 Hz, 2H), 2.44 (s, 3H), 1.35 (s, 9H), 1.31 (t, $J$ = 7.2 Hz, 3H); $^{13}C$ NMR ($CDCl_3$): δ 165.5, 150.6, 144.4, 137.0, 136.3, 129.3 (2C), 128.2 (2C), 124.8, 84.6, 61.0, 47.2, 27.8 (3C), 21.6, 14.2 ppm.

**Ethyl 2-[{(*N*-(tert-butoxycarbonyl)-2-nitrophenyl)sulfonamido}methyl]acrylate (13b)**

Acrylate **13b** was prepared by the procedure similar to that of **13a** with ethyl 2-(hydroxymethyl)acrylate (**12**) (1.95 g, 15 mmol). Purification by silica gel column chromatography with hexane/EtOAc (9/1) gave **13b** (5.78 g, 93%) as a colorless oil. IR (ATR): ν 1739, 1720, 1649, 1537, 795 $cm^{-1}$; $^1H$ NMR

($CDCl_3$): δ 8.38 (d, $J$ = 8.7 Hz, 1H), 7.78-7.76 (m, 3H), 6.42 (s, 1H), 5.89 (s, 1H), 4.67 (s, 2H), 4.26 (q, $J$ = 7.2 Hz, 2H), 1.33 (s, 9H), 1.30 (t, 3H) ppm; $^{13}$C NMR ($CDCl_3$): δ 165.3, 150.0, 147.7, 136.5, 134.4, 133.9, 133.2, 131.8, 125.0, 124.6, 85.3, 61.1, 48.0, 27.8 (3C), 14.2 ppm; HRMS(ESI)($m/z$) for $C_{17}H_{22}N_2NaO_8S$ ($MNa^+$): Calculated 437.0995 found 437.0981.

**Ethyl 2-[{(4-methylphenyl)sulfonamido}methyl]acrylate (14a)**

Tosylamide **14a** was prepared according to the literature procedure.[4] Acrylate **13a** (4.98 g, 13 mmol) was dissolved in $CH_2Cl_2$ (92 mL) under an argon atmosphere, and the solution was cooled to 0 ºC. TFA (38.5 mL) was slowly added, and the mixture was warmed up to room temperature. After stirring for 3 hours, the reaction solution was concentrated *in vacuo*. The resulting crude product was used in the next step without purification.

**Ethyl 2-[{(2-nitrophenyl)sulfonamido}methyl]acrylate (14b)**

Nosylamide **14b** was prepared by the procedure similar to that of **14a** with acrylate **13b** (5.78 g, 14 mmol). The resulting crude product was used in the next step without purification.

***N*-{2-(Hydroxymethyl)allyl}-4-methylbenzenesulfonamide (4aa)**

The crude product **14a** was dissolved in $CH_2Cl_2$ (130 mL) under an argon atmosphere, and the solution was cooled to -78 ºC. DIBAL-H (1.0 M in hexane, 52 mL, 52 mmol) was added dropwise over 20 min at -78 ºC. After stirring at the same temperature for 15 min, the reaction was quenched by addition of saturated $NH_4Cl$ aq. (50 mL) and saturated potassium sodium tartrate aq. (50 mL). The organic layer was separated, and the aqueous layer was extracted with EtOAc (50 mL) three times. The combined organic layer was washed with brine, dried over $Na_2SO_4$, and filtered through a cotton plug. The filtrate was concentrated *in vacuo*, and the residue was purified by silica gel column chromatography with hexane/EtOAc (10/1 to 5/1) to afford **4aa** (2.16 g, 69% yield in 2 steps) as slightly yellow oil. The $^1$H NMR spectrum was identical to that in the literature.[5] $^1$H NMR ($CDCl_3$): δ 7.75 (d, $J$ = 7.4 Hz, 2H), 7.32 (d, $J$ = 7.4 Hz, 2H), 5.10 (s, 1H), 5.03 (s, 1H), 4.65 (brs, 1H), 4.13 (d, $J$ = 5.9 Hz, 2H), 3.64 (d, $J$ = 6.4 Hz, 2H), 2.46 (s, 3H), 1.77 (t, $J$ = 6.4 Hz, 1H) ppm; $^{13}$C NMR ($CDCl_3$): δ 143.8, 143.7, 136.7, 129.8 (2C), 127.1 (2C), 114.3, 64.3, 45.8, 21.6 ppm.

***N*-{2-(Hydroxymethyl)allyl}-2-nitrobenzenesulfonamide (4ab)**

Allylnosylamide **4ab** was prepared by the procedure similar to that of **4aa** with the crude product **14b**. Purification by silica gel column chromatography with hexane/EtOAc (10/1 to 5/1) gave **4ab** (2.74 g, 72% in 2 steps) as a colorless oil. IR (ATR): ν 3506, 3234, 1699, 1540, 1165, 772 $cm^{-1}$. $^1$H NMR ($CDCl_3$): δ 8.14-8.12 (m, 1H), 7.88-7.86 (m, 1H), 7.76-7.73 (m, 2H), 5.65 (brs, 1H), 5.12 (s, 1H), 5.08 (s, 1H), 4.14 (d, $J$ = 4.7 Hz, 2H), 3.82 (d, $J$ = 6.3 Hz, 2H), 1.76 (brs, 1H) ppm. $^{13}$C NMR ($CDCl_3$): δ 148.1,

143.3, 133.7, 133.6, 132.8, 131.2, 125.4, 114.5, 64.2, 46.2 ppm. HRMS(ESI)(*m/z*) for $C_{10}H_{12}N_2NaO_5S$ ($MNa^+$): Calculated 295.0365, found 295.0336.

### 2.3 Cu-mediated *N*- and *O*-alkenylation

**Purification of copper iodide**

Copper iodide (CuI) was prepared according to the literature procedure.[6] CuI (5.4 g) and NaI aq. (15.2 g) was suspended in $H_2O$ (12 mL), and the suspension was stirred at reflux temperature for 2 hours. After cooling to room temperature, $H_2O$ (12 mL) was added to the suspension. The mixture was filtered and washed sequentially with $H_2O$ (12 mL), EtOH (12 mL), EtOAc (12 mL), $Et_2O$ (12 mL), and pentane (12 mL). After drying *in vacuo* over 8 hours, pure CuI was obtained (5.2 g).

**General procedure of Cu-mediated *N*- and *O*-alkenylation (Table 1 and 2)**

Purified CuI (38 mg, 0.20 mmol) was suspended in anhydrous solvent (0.10 mL) under an argon atmosphere. Under the conditions in Table 1, *N,N'*-dimethylethylenediamine (45 µL, 0.40 mmol) was added to the suspension. To the solution, sulfonamide **4aa** or **4ab** （0.20 mmol） in anhydrous solvent (0.10 mL), base (0.50 mmol), and vinyl iodide **3a** (83 mg, 0.40 mmol) were added at 0 ºC. The reaction mixture was stirred under heating for 19-24 hours. The reaction mixture was cooled to room temperature and quenched by addition of saturated $NH_4Cl$ aq. (0.5 mL). The organic layer was separated, and the aqueous layer was extracted with EtOAc (1.0 mL) three times. The combined organic layer was washed with brine, dried over $Na_2SO_4$, and filtered through a cotton plug. The filtrate was concentrated *in vacuo*, and the residue was purified by silica gel column chromatography with hexane/EtOAc (10/1 to 5/1) to isolate **5**, **8** and **9**.

***N*-(Cyclohex-1-en-1-yl)-*N*-{2-(hydroxymethyl)allyl}-4-methylbenzenesulfonamide (5a)**: pale yellow oil; IR (ATR): ν 3200, 1773, 1699, 1160, 772 $cm^{-1}$; $^1H$ NMR ($CDCl_3$): δ 7.68 (d, *J* = 8.1 Hz, 2H), 7.28 (d, *J* = 8.1 Hz, 2H), 5.50 (brs, 1H), 5.11 (s, 1H), 4.95 (s, 1H), 4.20 (s, 2H), 3.95 (s, 2H), 2.43 (s, 3H), 2.38 (brs, 1H), 2.05-2.03 (m, 2H), 1.91 (brs, 2H), 1.58-1.55 (m, 2H), 1.52-1.47 (m, 2H) ppm; $^{13}C$ NMR ($CDCl_3$): δ 144.1, 143.4, 136.3, 136.3, 129.5 (2C), 129.3, 127.5 (2C), 114.8, 63.4, 51.4, 28.1, 25.1, 22.9, 21.6, 21.5 ppm; HRMS(ESI)(*m/z*) for $C_{17}H_{23}NNaO_3S$ ($MNa^+$): Calculated 344.1284, found 344.1296.

***N*-[2-{(2-Oxocyclohexyl)methyl}allyl]-4-methylbenzenesulfonamide (8a)**: yellow oil; IR (ATR): ν 3282, 1703, 1599, 1159, 755 $cm^{-1}$; $^1H$ NMR ($CDCl_3$): δ 7.74 (d, *J* = 8.1 Hz, 2H), 7.29 (d, *J* = 8.0 Hz, 2H), 4.94 (s, 1H), 4.84 (t, *J* = 6.1 Hz, 1H), 4.79 (s, 1H), 3.50 (dd, *J* = 14.4, 6.2 Hz, 1H), 3.45 (dd, *J* = 14.5, 6.2 Hz, 1H), 2.46-2.36 (m, 3H), 2.42 (s, 3H), 2.31-2.24 (m, 1H), 2.08-2.01 (m, 2H), 1.89-1.84 (m, 2H), 1.68-1.59 (m, 2H), 1.32-1.27 (m, 1H) ppm; $^{13}C$ NMR ($CDCl_3$): δ 212.7, 143.4, 142.5, 137.0, 129.7 (2C), 127.2 (2C), 114.1, 49.0, 48.2, 42.1, 34.0, 33.2, 28.1, 25.0, 21.5 ppm; HRMS(ESI)(*m/z*) for $C_{17}H_{23}NNaO_3S$ ($MNa^+$): Calculated 344.1296, found 344.1269.

***N*-[2-{2-(Hydroxymethyl)allyl}cyclohexylidene]-4-methylbenzenesulfonamide (9a)**: pale yellow oil; IR (ATR): ν 3288, 1723, 1597, 1156, 772 $cm^{-1}$; $^1H$ NMR ($CDCl_3$): δ 7.87 (d, *J* = 7.6 Hz, 2H), 7.31 (d, *J* = 7.6

Hz, 2H). 4.98 (s, 1H), 4.97 (s, 1H), 4.03 (d, $J$ = 13.1 Hz, 1H), 3.97 (d, $J$ = 13.4 Hz, 1H), 2.79-2.64 (m, 4H), 2.42 (s, 3H), 2.36-2.29 (m, 1H), 2.09-2.04 (m, 1H), 1.97-1.94 (m, 2H), 1.88-1.80 (m,1H), 1.62-1.59 (m, 1H), 0.89-0.87 (m, 1H) ppm; $^{13}$C NMR ($CDCl_3$): δ 207.2, 143.1, 140.7, 137.9, 129.3 (2C), 127.7 (2C), 109.0, 75.8, 52.2, 44.7, 40.0, 37.3, 24.5, 23.5, 21.5 ppm; HRMS(ESI)(*m/z*) for $C_{17}H_{23}NNaO_3S$ ($MNa^+$): Calculated 344.1303, found 344.1230.

***N*-[2-{(2-Oxocyclohexyl)methyl}allyl]-2-nitrobenzenesulfonamide (8b)**: yellow oil; IR (ATR): ν 3456, 1766, 1704, 1541, 1167, 771 cm$^{-1}$; $^1$H NMR ($CDCl_3$): δ 8.10 (dd, $J$ = 9.2, 3.4 Hz, 1H), 7.85 (dd, $J$ = 9.2, 3.4 Hz, 1H), 7.72 (m, 2H), 5.70 (brs, 1H), 5.00 (s, 1H), 4.81 (s, 1H), 3.68 (dd, $J$ = 15.0, 6.4 Hz, 2H), 2.49-2.46 (m, 2H), 2.41-2.38 (m, 1H), 2.33-2.26 (m, 1H), 2.07-2.05 (m, 1H), 2.04 (brs, 1H), 1.91-1.84 (m, 2H), 1.68-1.60 (m, 2H), 1.34-1.27 (m, 1H) ppm; $^{13}$C NMR ($CDCl_3$): δ 212.3, 148.1, 142.2, 134.1, 133.5, 132.7, 131.0, 125.3, 114.2, 49.1, 48.7, 42.1, 34.0, 33.1, 28.0, 25.0 ppm; HRMS(ESI)(*m/z*) for $C_{16}H_{20}N_2NaO_5S$ ($MNa^+$): Calculated 375.0991, found 375.1000.

## 2.4 Conversion to hydroquinoline 6

### 3-Methylene-1-tosyl-1,2,3,4,5,6,7,8-octahydroquinoline (6a)

In a glove box, enamide **5a** (32 mg, 0.10 mmol) was dissolved in 1,2-dichloroethane (1.0 mL) in a glass vial under nitrogen atmosphere, and $AgSbF_6$ (3.4 mg, 10 mol%) was added to the solution. The glass vial was sealed by a cap and taken out from the glove box. The reaction mixture was stirred at 120 ºC for 1 hour by using Biotage microwave synthesizer. After cooling to room temperature, saturated $NH_4Cl$ aq. (2.0 mL) was added to the mixture at 0 ºC. The organic layer was separated, and the aqueous layer was extracted with $CH_2Cl_2$ (2.0 mL) three times. The combined organic layer was washed with brine, dried over $Na_2SO_4$, and filtered through a cotton plug. The filtrate was concentrated *in vacuo*, and the residue was purified by silica gel column chromatography with hexane/EtOAc (20/1 to 10/1) to afford hydroquinoline **6a** (7.9 mg, 26% yield) as slightly yellow oil. IR (ATR): ν 1653, 1597, 1159, 777 cm$^{-1}$; $^1$H NMR ($CDCl_3$): δ 7.58 (d, $J$ = 7.6 Hz, 2H), 7.18 (d, $J$ = 7.6 Hz, 2H), 4.71 (s, 1H), 4.54 (s, 1H), 4.04 (s, 2H), 2.51 (brs, 2H), 2.39 (s, 3H), 2.20 (s, 2H), 1.90 (brs, 2H), 1.67-1.66 (m, 2H), 1.62-1.61 (m, 2H) ppm; $^{13}$C NMR ($CDCl_3$): δ 143.0, 139.1, 135.9, 131.7, 128.9 (2C), 127.9 (2C), 126.7, 110.6, 53.0, 34.5, 30.0, 30.0, 23.5, 22.4, 21.6 ppm; HRMS(ESI)(*m/z*) for $C_{17}H_{21}NNaO_2S$ ($MNa^+$): Calculated 375.0991, found 375.1000.

### 1-{3-Methylene-3,4,5,6,7,8-hexahydroquinolin-1(*2H*)-yl}ethan-1-one (6b)

Ketone **8b** (35 mg, 0.10 mmol) was dissolved in anhydrous acetonitrile (1.0 mL) under an argon atmosphere. $K_2CO_3$ (69 mg, 0.50 mmol) and thiophenol (51 μL, 0.50 mmol) were added to the solution, and the reaction mixture was stirred at room temperature. After 2.5 hours, the mixture was filtered through a small pad of silica gel, and the filtrate was concentrated *in vacuo*. The residue was dissolved in anhydrous diethyl ether (1.0 mL) under an argon atmosphere, and triethylamine (115 μL, 0.80 mmol) and acetic anhydride (61.5 μL, 0.65 mmol) were added. The reaction mixture was stirred at room temperature for 2 hours. The reaction

was quenched by addition of water (2.0 mL) at 0 ºC, and the organic layer was separated. The aqueous layer was extracted with EtOAc (2.0 mL) twice. The combined organic layer was washed with brine, dried over $Na_2SO_4$, and filtered through a cotton plug. The filtrate was concentrated *in vacuo*, and the residue was purified by silica gel column chromatography with hexane/EtOAc (10/1 to 5/1) to afford **6b** (18 mg, 96% yield) as slightly yellow oil. IR (ATR): ν 1652 $cm^{-1}$; $^1H$ NMR ($CDCl_3$): δ 4.87 (s, 1H), 4.82 (s, 1H), 4.09 (brs, 1H), 3.98 (brs, 1H), 2.71 (s, 2H), 2.34 (brs, 2H), 2.09 (s, 3H), 2.06 (brs, 2H), 1.68-1.65 (m, 4H) ppm; $^{13}C$ NMR ($CDCl_3$): δ 169.7, 142.2, 130.0, 108.6 (2C), 53.2 (rotamer), 48.7 (rotamer), 35.8, 32.7, 29.7 (rotamer), 29.3, 28.2 (rotamer), 23.4, 22.5 ppm; HRMS(ESI)(*m*/*z*) for $C_{12}H_{17}NNaO$ ($MNa^+$): Calculated 214.1208, found 214.1202. In $^{13}C$ NMR spectrum of **6b**, the signals were broadened and the signals of C2 and C8 of the hydroquinoline skeleton were split (53.2, 48.7, 29.7, 28.2 ppm) owing to the rotamers of amide.

## 3. $^{1}$H and $^{13}$C NMR Spectra

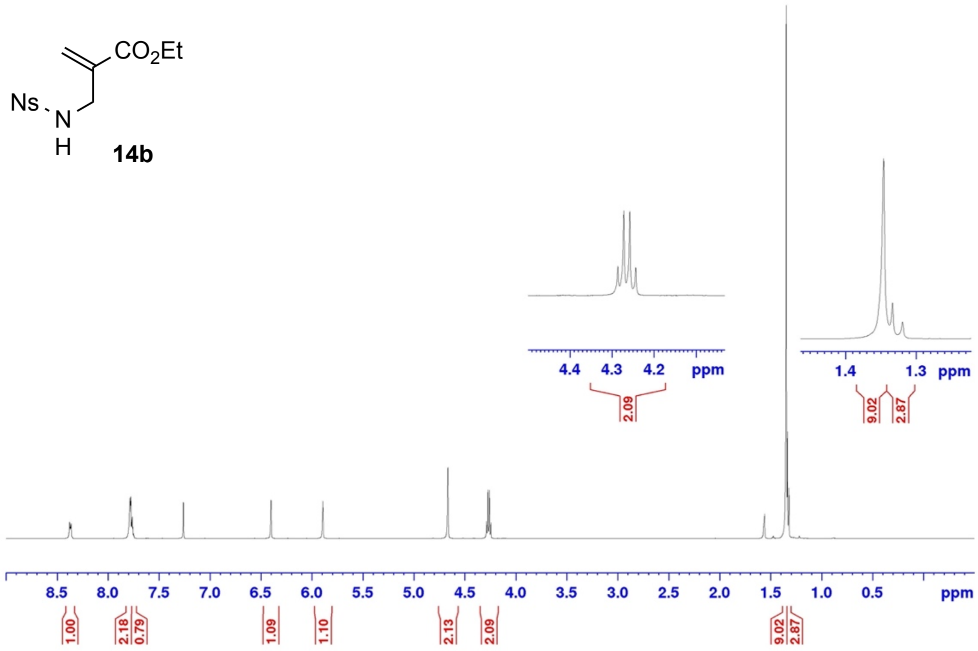


**Figure S1.** $^{1}$H NMR (500 MHz, $CDCl_3$) spectrum of compound **14b**

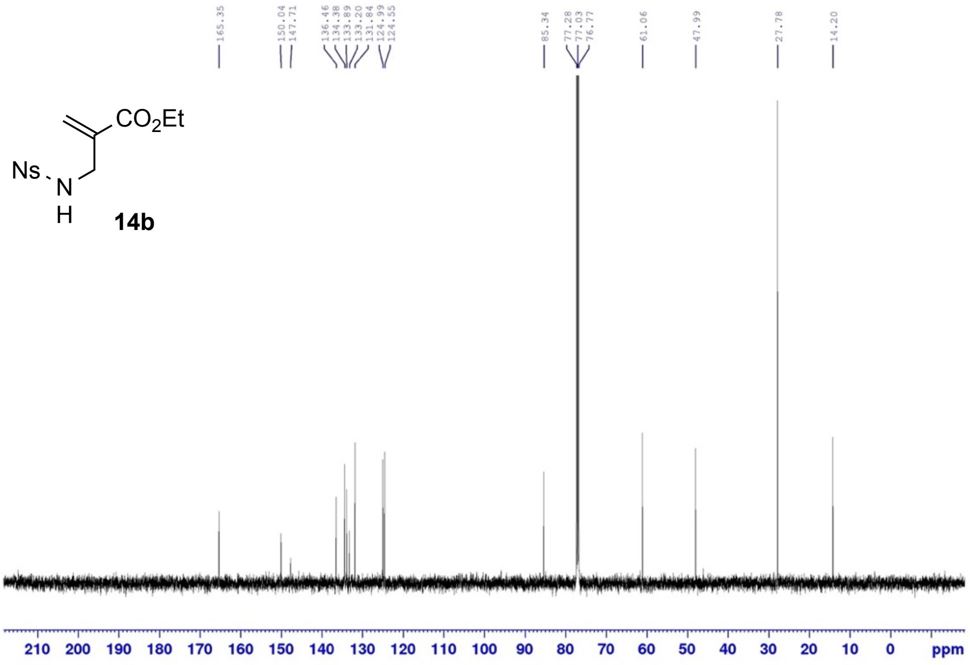


**Figure S2.** $^{13}$C NMR (125 MHz, $CDCl_3$) spectrum of compound **14b**

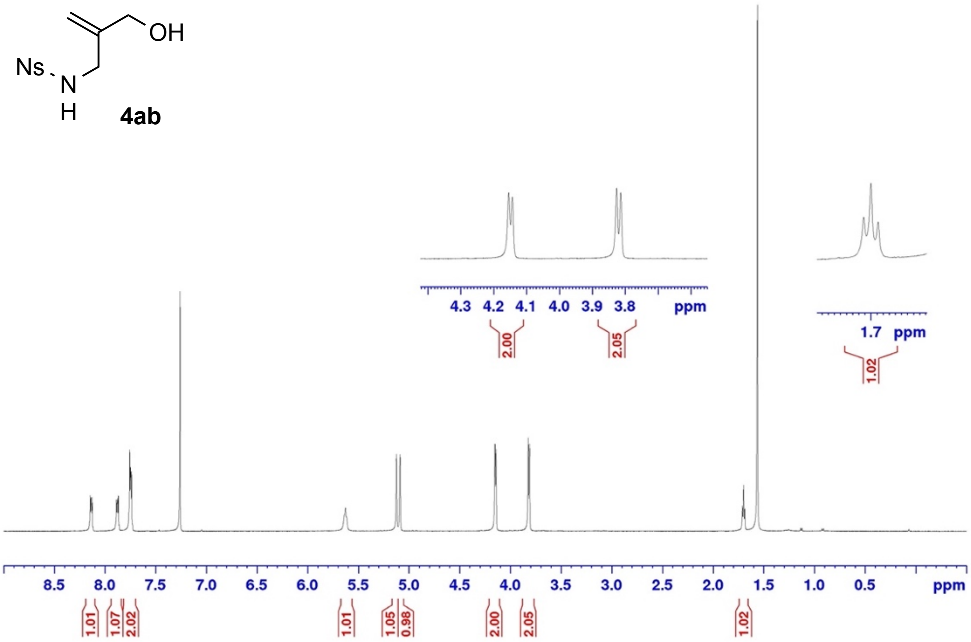


**Figure S3.** $^{1}$H NMR (500 MHz, $CDCl_3$) spectrum of compound **4ab**

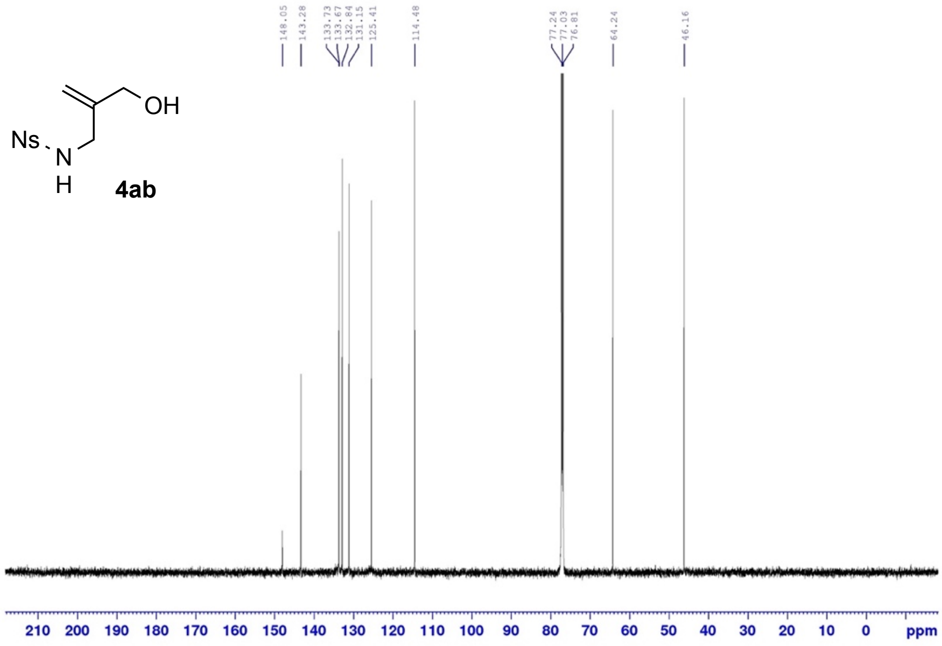


**Figure S4.** $^{13}$C NMR (125 MHz, $CDCl_3$) spectrum of compound **4ab**

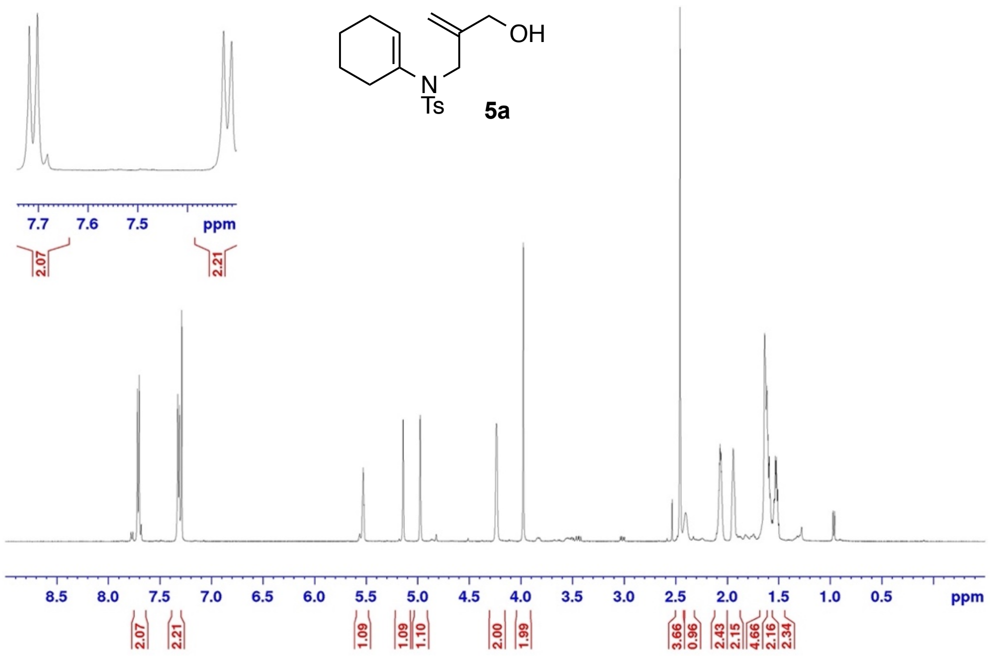

**Figure S5.** $^{1}H$ NMR (500 MHz, $CDCl_3$) spectrum of compound **5a**

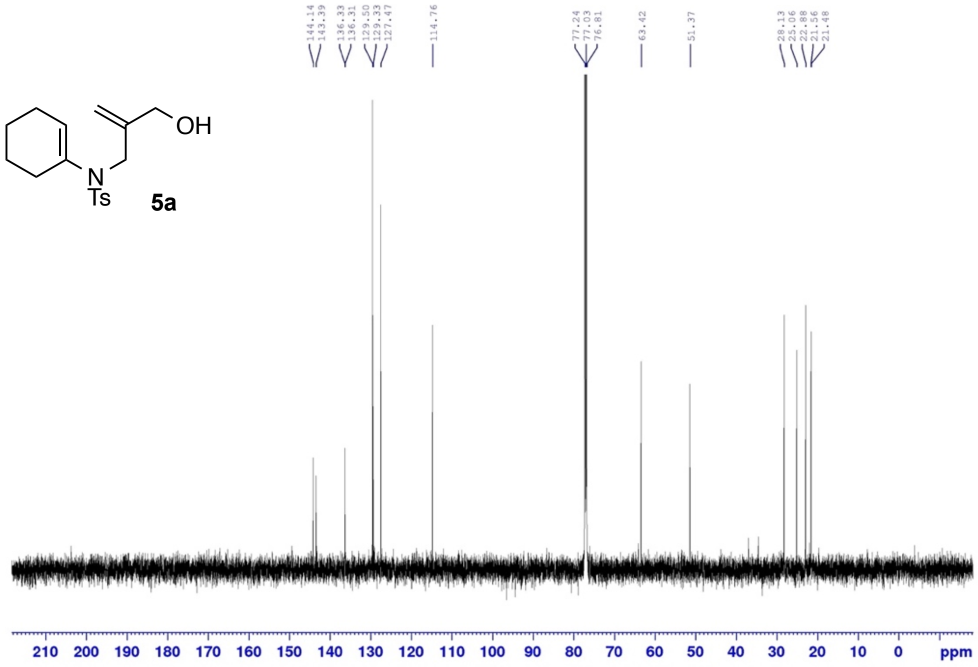

**Figure S6.** $^{13}C$ NMR (125 MHz, $CDCl_3$) spectrum of compound **5a**

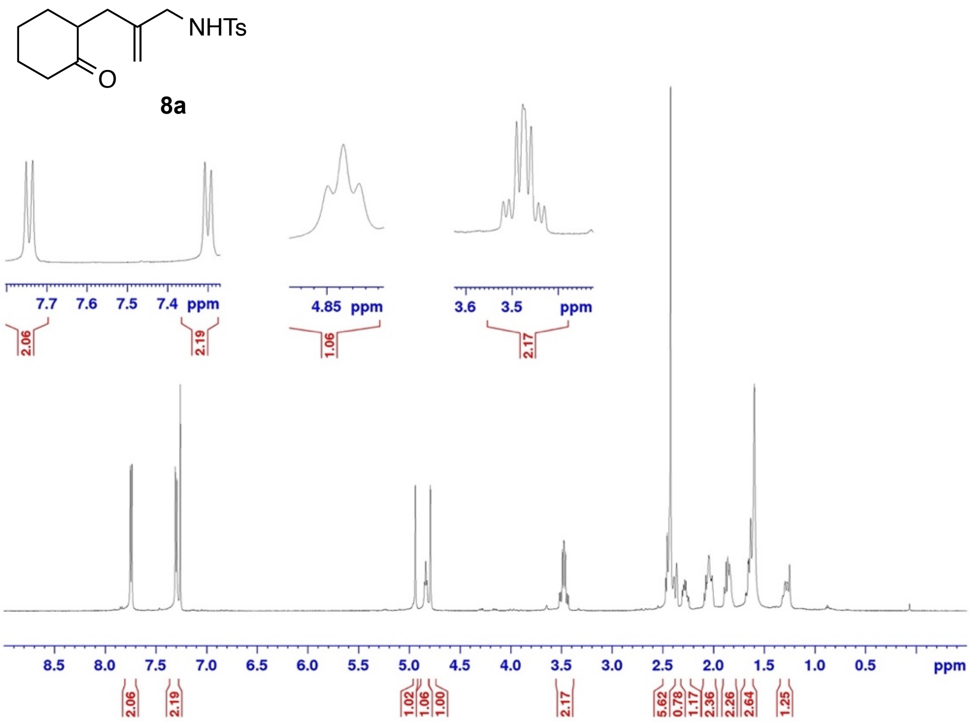


**Figure S7.** $^{1}$H NMR (500 MHz, $CDCl_3$) spectrum of compound **8a**

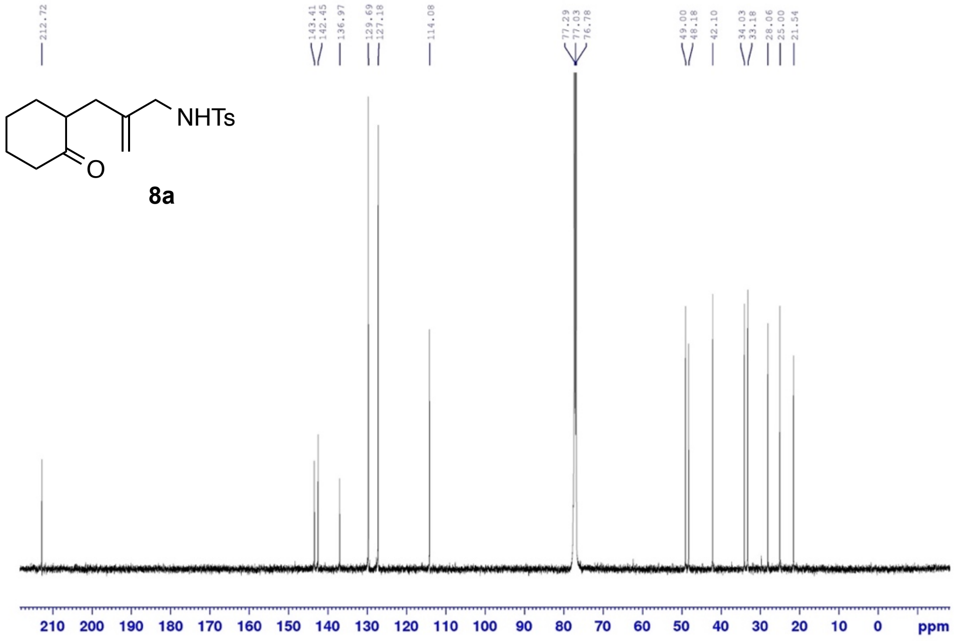


**Figure S8.** $^{13}$C NMR (125 MHz, $CDCl_3$) spectrum of compound **8a**

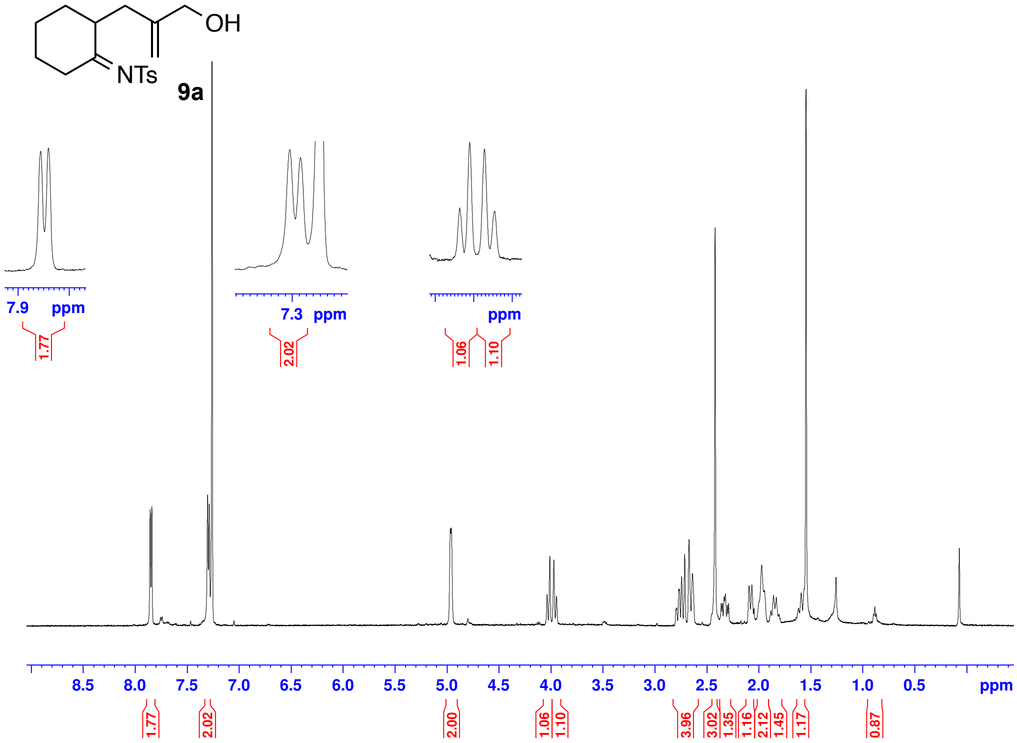


**Figure S9.** $^{1}H$ NMR (500 MHz, $CDCl_3$) spectrum of compound **9a**

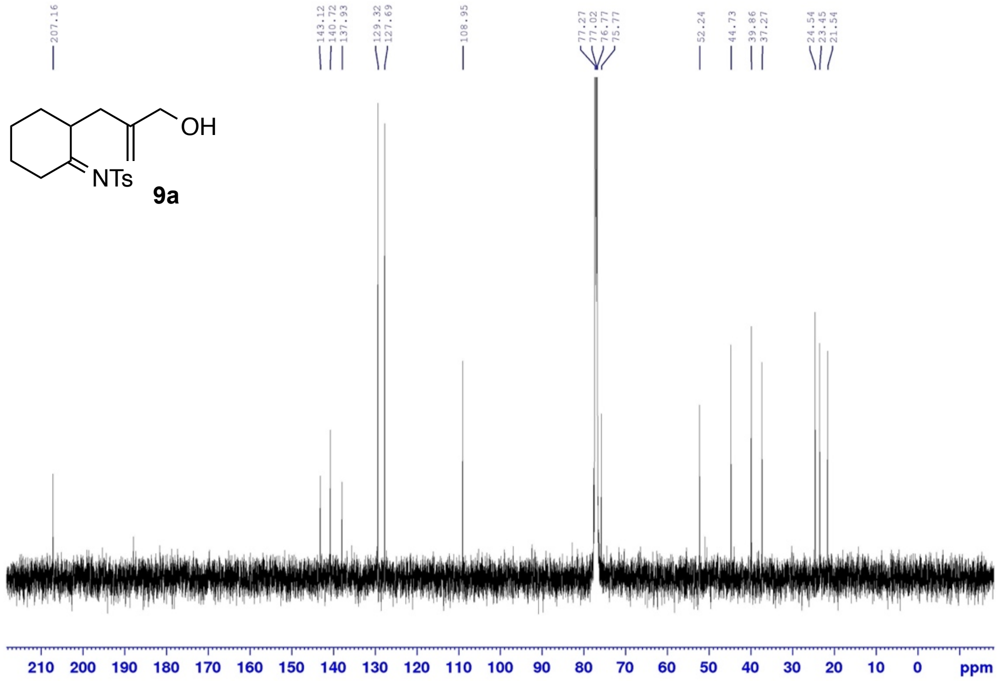


**Figure S10.** $^{13}C$ NMR (125 MHz, $CDCl_3$) spectrum of compound **9a**

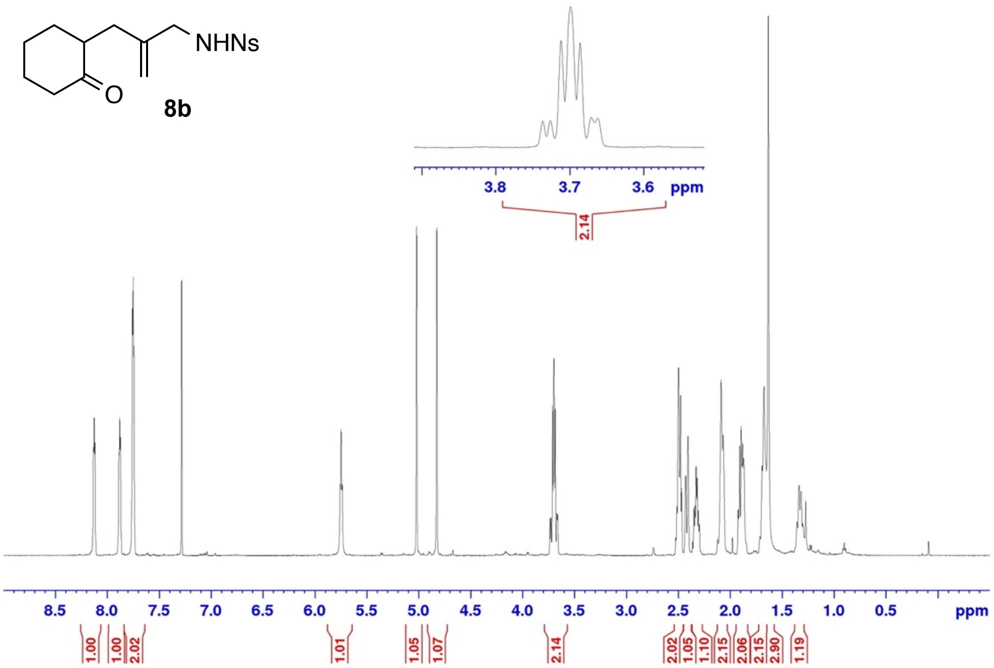


**Figure S11.** $^{1}H$ NMR (500 MHz, $CDCl_3$) spectrum of compound **8b**

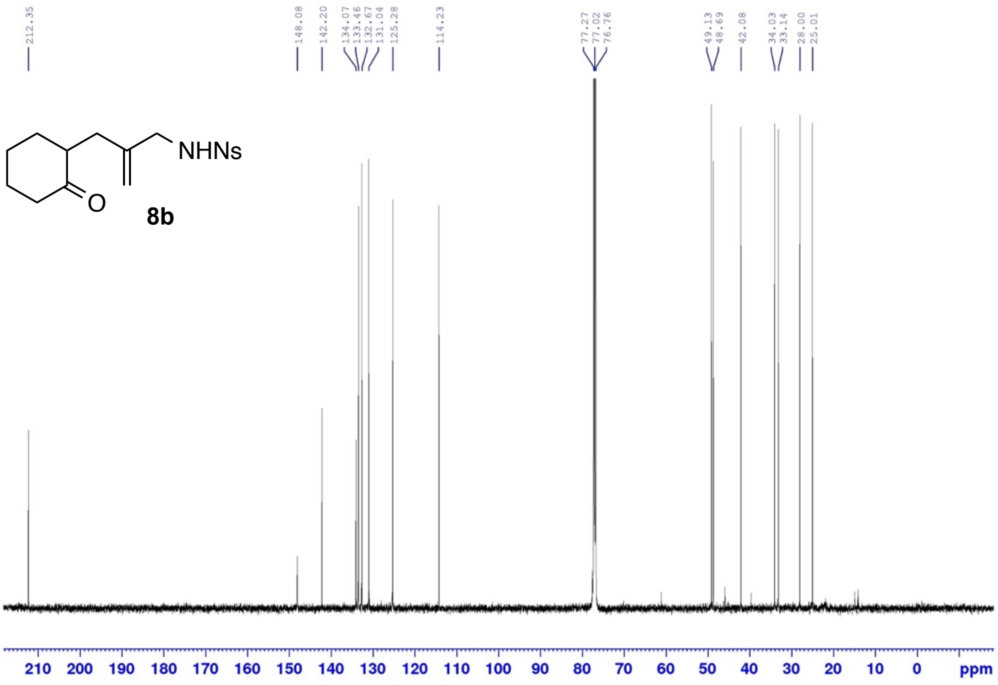


**Figure S12.** $^{13}C$ NMR (125 MHz, $CDCl_3$) spectrum of compound **8b**

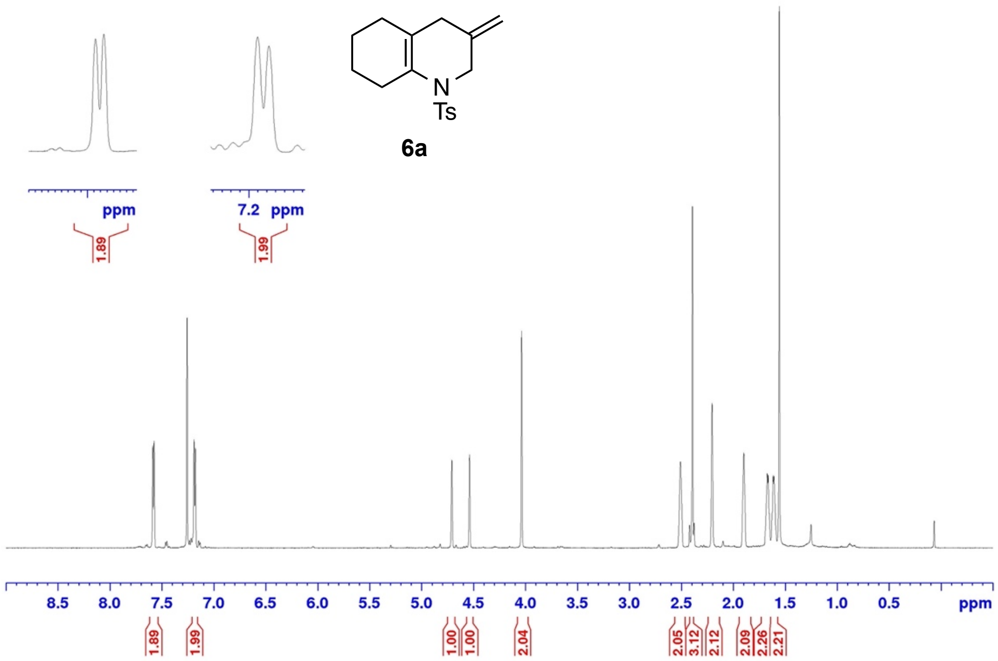


**Figure S13.** $^{1}$H NMR (500 MHz, $CDCl_3$) spectrum of compound **6a**

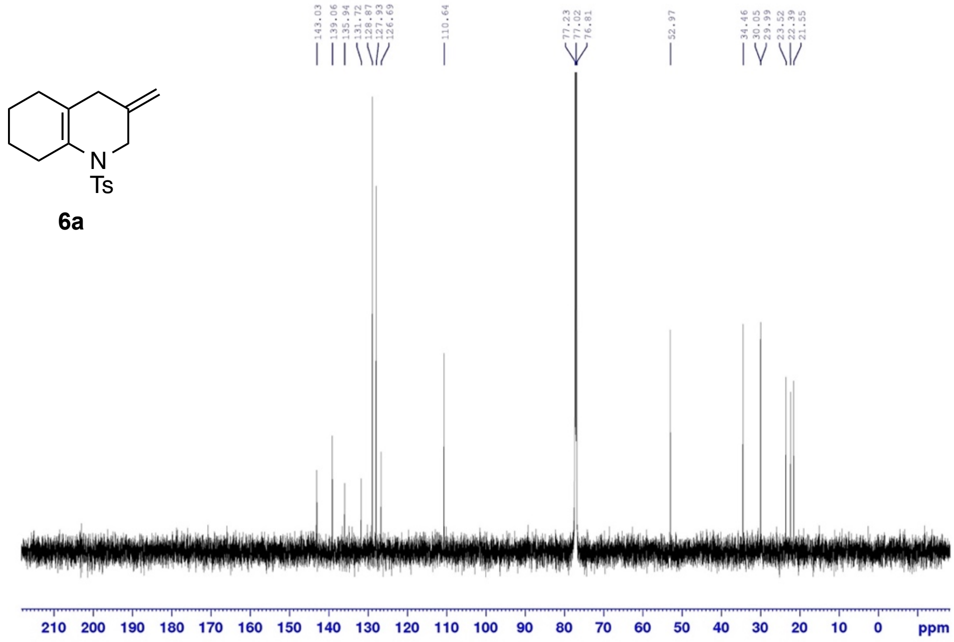


**Figure S14.** $^{13}$C NMR (125 MHz, $CDCl_3$) spectrum of compound **6a**

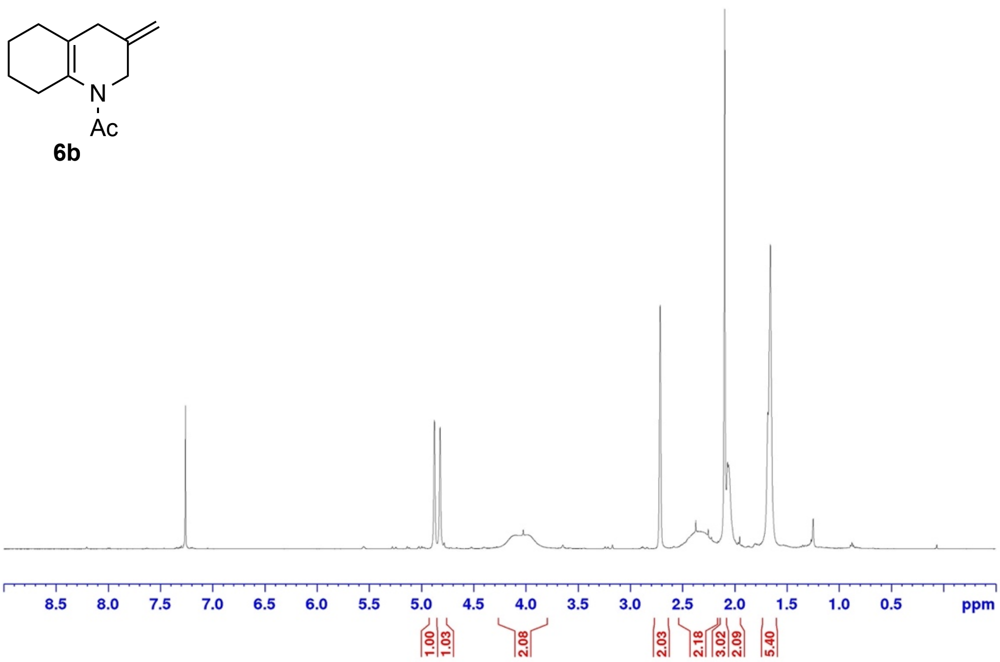


**Figure S15.** $^{1}$H NMR (500 MHz, $CDCl_3$) spectrum of compound **6b**

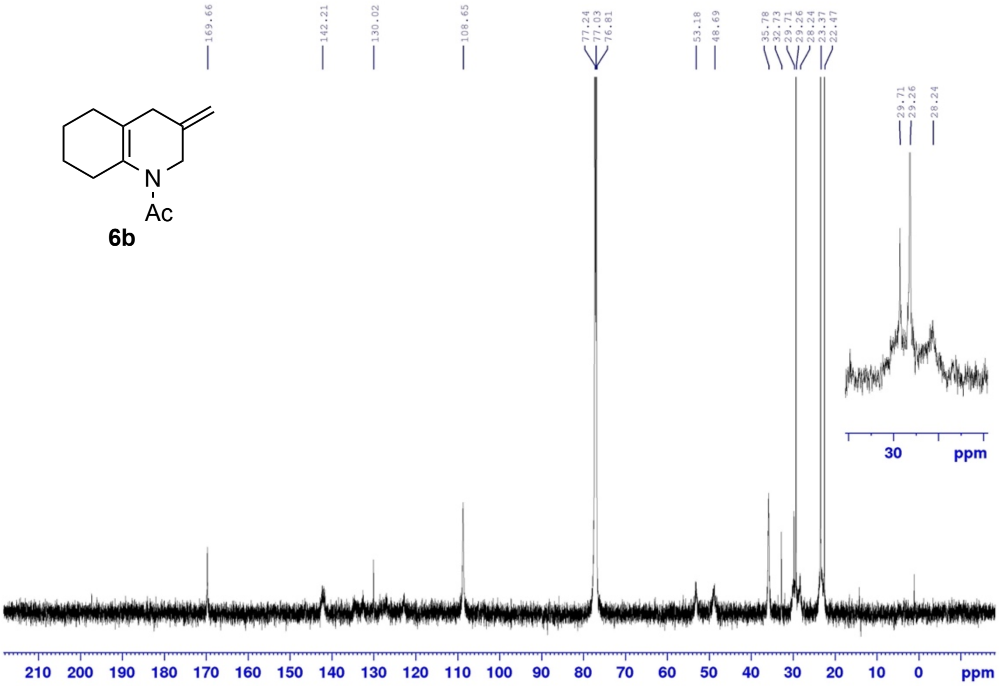


**Figure S16.** $^{13}$C NMR (125 MHz, $CDCl_3$) spectrum of compound **6b**

## 4. Theoretical Calculation

DFT calculations was performed at PBE0/6-31+G(d) level of theory by using the Gaussian 16 program package.[7)] The structures were optimized and the vibrational frequency analyses were conducted on the optimized structures. The given energies are zero-point corrected.

**7**

E = -1299.344069 a.u.

| | | | |
|---|---|---|---|
| C | -2.65982 | -1.07788 | -0.3838 |
| C | -2.1675 | 0.14407 | -1.10687 |
| H | -1.21626 | 0.48691 | -0.67371 |
| H | -1.95611 | -0.11344 | -2.15406 |
| C | -3.19842 | 1.27315 | -1.05894 |
| C | -3.78266 | 1.42844 | 0.34058 |
| H | -2.73182 | 2.20736 | -1.39473 |
| H | -4.01123 | 1.05049 | -1.76454 |
| H | -2.96688 | 1.628 | 1.04992 |
| H | -4.46274 | 2.28858 | 0.38201 |
| C | -3.49545 | -4.09764 | 0.18311 |
| C | -2.19218 | -3.3479 | 0.2671 |
| H | -2.02855 | -3.01484 | 1.30061 |
| H | -1.37031 | -4.03664 | 0.02746 |
| C | -3.60392 | -5.28701 | 1.10194 |
| H | -4.62065 | -5.69919 | 1.05102 |
| H | -3.42888 | -4.98168 | 2.13952 |
| O | -2.05808 | -2.28691 | -0.65204 |
| N | -2.70483 | -6.38933 | 0.72972 |
| H | -2.13121 | -6.21235 | -0.0895 |
| S | -3.33469 | -7.93632 | 0.55173 |
| O | -2.53226 | -8.53244 | -0.51325 |
| O | -4.7903 | -7.86029 | 0.47329 |
| C | -2.93431 | -8.78182 | 2.06792 |
| C | -1.66457 | -9.33793 | 2.21415 |
| C | -1.36222 | -10.02558 | 3.38556 |
| H | -0.93832 | -9.24834 | 1.41171 |
| C | -3.59049 | -9.59892 | 4.22491 |
| C | -2.32344 | -10.15565 | 4.38878 |
| H | -0.37875 | -10.47088 | 3.51018 |
| H | -4.34096 | -9.70889 | 5.00311 |
| H | -2.0851 | -10.69985 | 5.29913 |
| C | -3.71562 | -1.07658 | 0.43966 |
| H | -4.01773 | -2.02269 | 0.88589 |
| C | -3.90498 | -8.90635 | 3.05752 |
| H | -4.89064 | -8.47903 | 2.9032 |
| C | -4.46876 | -3.78851 | -0.67579 |
| H | -5.38817 | -4.36899 | -0.71023 |
| H | -4.37217 | -2.95441 | -1.36379 |
| C | -4.51429 | 0.15452 | 0.75612 |
| H | -5.49385 | 0.10686 | 0.25289 |
| H | -4.73764 | 0.18285 | 1.83156 |

TS$_{7\text{-}8}$

E = -1299.306655 a.u.

| | | | |
|---|---|---|---|
| C | 2.77655 | 0.35396 | -0.61929 |
| C | 4.12103 | 0.79245 | -0.96442 |
| H | 4.41396 | 0.26688 | -1.89556 |
| H | 4.07097 | 1.86942 | -1.21626 |
| C | 5.18296 | 0.54634 | 0.11727 |
| C | 5.08144 | -0.88638 | 0.65683 |
| H | 6.18932 | 0.72385 | -0.30312 |
| H | 5.07412 | 1.27547 | 0.94426 |
| H | 5.29819 | -1.6079 | -0.15389 |
| H | 5.84951 | -1.05569 | 1.43474 |
| C | 1.03598 | -0.95733 | -1.29119 |
| C | 1.31055 | -0.01181 | -2.21861 |
| H | 2.14295 | -0.07799 | -2.93118 |
| H | 0.46294 | 0.51637 | -2.68237 |
| C | -0.39918 | -1.1127 | -0.85423 |
| H | -0.67331 | -2.19295 | -0.80501 |
| H | -1.08775 | -0.64044 | -1.59646 |
| C | -3.02205 | 0.70193 | 0.4189 |
| H | 1.56748 | -0.93503 | 0.88232 |
| O | 1.83116 | 0.992 | -1.08652 |
| S | -2.12165 | -0.53888 | 1.23273 |
| H | 0.18908 | 0.04452 | 0.84163 |
| O | -2.72337 | -1.82081 | 0.91998 |
| O | -1.88137 | -0.14005 | 2.60542 |
| C | -2.8433 | 2.04148 | 0.77346 |
| H | -5.30924 | 1.08653 | -2.07656 |
| H | -2.15326 | 2.31327 | 1.5787 |
| C | -4.61799 | 1.35401 | -1.27956 |
| C | -4.44109 | 2.69593 | -0.92344 |
| C | -3.55356 | 3.04156 | 0.10109 |
| H | -3.41535 | 4.08579 | 0.37638 |
| H | -4.99849 | 3.47448 | -1.44523 |
| C | 2.55033 | -0.71927 | 0.44891 |
| N | -0.61283 | -0.47867 | 0.46989 |
| C | -3.90758 | 0.35651 | -0.60557 |
| H | -4.05093 | -0.69592 | -0.87561 |
| C | 2.16805 | -1.77413 | -0.67613 |
| H | 2.02376 | -2.51997 | 0.09387 |
| H | 3.17525 | -1.75706 | -1.08294 |
| C | 3.6914 | -1.131 | 1.25573 |
| H | 3.56183 | -0.50382 | 2.16506 |
| H | 3.60443 | -2.18124 | 1.59718 |

**8**

E = -1299.377588 a.u.

| | | | |
|---|---|---|---|
| C | 2.82375 | 1.0379 | -0.62292 |
| C | 4.26993 | 0.88207 | -1.03327 |
| H | 4.40879 | -0.14888 | -1.39302 |
| H | 4.4657 | 1.5543 | -1.87398 |
| C | 5.21569 | 1.13788 | 0.14827 |
| C | 4.84645 | 0.27733 | 1.35381 |
| H | 6.25043 | 0.94825 | -0.16146 |
| H | 5.16277 | 2.19989 | 0.42717 |
| H | 5.01406 | -0.78221 | 1.11547 |
| H | 5.50795 | 0.50836 | 2.19789 |
| C | 1.30715 | -1.54144 | -0.84511 |
| C | 1.6375 | -1.2253 | -2.10024 |
| H | 2.56999 | -0.71501 | -2.33302 |
| H | 0.993 | -1.47118 | -2.94076 |
| C | -0.01385 | -2.20763 | -0.54596 |
| H | -0.05829 | -2.45775 | 0.51666 |
| H | -0.10186 | -3.14173 | -1.1218 |
| C | 2.39216 | 0.27518 | 0.62203 |
| H | 1.41868 | 0.69516 | 0.90637 |

| | | | | | | | |
|---|---|---|---|---|---|---|---|
| O | 2.04415 | 1.73942 | -1.2387 | H | -5.0566 | -4.56836 | -2.99435 |
| N | -1.13688 | -1.30504 | -0.82009 | H | -3.00972 | -6.42363 | 0.30205 |
| H | -1.31656 | -1.12769 | -1.8046 | H | -4.41243 | -6.60843 | -1.73618 |
| S | -2.58399 | -1.5508 | -0.03213 | C | -2.83626 | -4.28472 | 0.17872 |
| O | -3.4834 | -0.54207 | -0.57752 | H | -2.24232 | -4.18401 | 1.08228 |
| O | -2.26981 | -1.64308 | 1.38691 | C | 2.2162 | -1.22992 | 0.31546 |
| C | -3.1922 | -3.14952 | -0.5472 | H | 3.19113 | -1.69606 | 0.13527 |
| C | -3.98949 | -3.23674 | -1.68789 | H | 1.82079 | -1.7045 | 1.22668 |
| C | -4.427 | -4.48823 | -2.11218 | C | 3.39346 | 0.5067 | 1.76926 |
| H | -4.27884 | -2.33128 | -2.21395 | H | 3.12734 | -0.13869 | 2.61724 |
| C | -3.27833 | -5.53163 | -0.25737 | H | 3.28723 | 1.54233 | 2.1217 |
| C | -4.06807 | -5.63346 | -1.40131 | | | | |

## 5. References


1. T. Calogeropoulou, G. B. Hammond, D. F. Wieme, *J. Org. Chem.* **1987**, *52*, 4185.
2. K. Lee, D. F. Wiemer, *Tetrahedron Lett.* **1993**, *34*, 2433.
3. J. J. Mousseau, J. A. Bull, A. B. Charette, *Angew. Chem. Int. Ed.* **2010**, *49*, 1115.
4. Žukauskaitė, S. Mangelinckx, G. Callebaut, C. Wybon, A. Šačkus, N. De Kimpe, *Tetrahedron*, **2013**, *69*, 3437.
5. H. Tsukamoto, A. Kawase, T. Doi, *Adv. Synth. Catal.* **2019**, *361*, 3733.
6. R. K. Dieter, L. A. Silks, J. R. Fishpaugh, M. E. Kastner, *J. Am. Chem. Soc.* **1985**, *107*, 4679.
7. Gaussian 16, Revision A.03, M. J. Frisch, G. W. Trucks, H. B. Schlegel, G. E. Scuseria, M. A. Robb, J. R. Cheeseman, G. Scalmani, V. Barone, G. A. Petersson, H. Nakatsuji, X. Li, M. Caricato, A. V. Marenich, J. Bloino, B. G. Janesko, R. Gomperts, B. Mennucci, H. P. Hratchian, J. V. Ortiz, A. F. Izmaylov, J. L. Sonnenberg, D. Williams-Young, F. Ding, F. Lipparini, F. Egidi, J. Goings, B. Peng, A. Petrone, T. Henderson, D. Ranasinghe, V. G. Zakrzewski, J. Gao, N. Rega, G. Zheng, W. Liang, M. Hada, M. Ehara, K. Toyota, R. Fukuda, J. Hasegawa, M. Ishida, T. Nakajima, Y. Honda, O. Kitao, H. Nakai, T. Vreven, K. Throssell, J. A. Montgomery, Jr., J. E. Peralta, F. Ogliaro, M. J. Bearpark, J. J. Heyd, E. N. Brothers, K. N. Kudin, V. N. Staroverov, T. A. Keith, R. Kobayashi, J. Normand, K. Raghavachari, A. P. Rendell, J. C. Burant, S. S. Iyengar, J. Tomasi, M. Cossi, J. M. Millam, M. Klene, C. Adamo, R. Cammi, J. W. Ochterski, R. L. Martin, K. Morokuma, O. Farkas, J. B. Foresman, and D. J. Fox, Gaussian, Inc., Wallingford CT, 2016.
